# Technical details of video‐assisted transcervical mediastinal dissection for esophageal cancer and its perioperative outcome

**DOI:** 10.1002/ags3.12022

**Published:** 2017-08-14

**Authors:** Kazuhiko Mori, Susumu Aikou, Koichi Yagi, Masato Nishida, Takashi Mitsui, Yukinori Yamagata, Hiroharu Yamashita, Sachiyo Nomura, Yasuyuki Seto

**Affiliations:** ^1^ Department of Gastrointestinal Surgery Graduate School of Medicine University of Tokyo Tokyo Japan; ^2^ Department of Gastrointestinal Surgery Mitsui Memorial Hospital Tokyo Japan; ^3^ Department of Surgery Dokkyo Medical University Koshigaya Hospital Koshigaya Japan

**Keywords:** cervical, esophageal cancer, lymphadenectomy, recurrent laryngeal nerve, video‐assisted surgery

## Abstract

To reduce pulmonary complications after esophagectomy, the transthoracic procedure should be shortened or totally avoided. Transcervical approach assisted by mediastinoscope for the upper mediastinum may be advantageous for this purpose. We carried out video‐assisted transcervical mediastinal dissection (VATCMD) as part of totally non‐transthoracic radical esophagectomy. A single‐port laparoscopy device was adopted to a small cervical incision and the mediastinum was inflated with a positive pressure of 6 to 10 mmHg. Without assistant's retractor, the upper mediastinum and partially the middle mediastinum were dissected mainly by mediastinoscopic‐assisted surgery. Video of the operation is demonstrated with illustrations. We have carried out and reported 17 cases of esophagectomy including VATCMD and its perioperative outcome. Non‐transthoracic esophagectomy was completed without conversion to transthoracic procedure in all 17 cases. Procedure‐related adverse event was not observed and postoperative course was favorable with a zero occurrence (0%) of recurrent laryngeal nerve palsy, chyle leakage or pulmonary complications. Median number of harvested lymph nodes from the upper mediastinal stations was 10. VATCMD is suggested as a safe and feasible approach for the upper mediastinum in esophagectomy for malignancies. It enabled a totally non‐transthoracic radical esophagectomy in combination with a transhiatal approach.

Video‐assisted transcervical mediastinal dissection is suggested as a safe and feasible approach for the upper mediastinum in esophagectomy for malignancies. It enabled a totally non‐transthoracic radical esophagectomy in combination with a transhiatal approach.

## 1 INTRODUCTION

Progress has been made in the surgical technique of esophagectomy and its surgical mortality has drastically decreased.^1^, ^2^, ^3^ Minimally invasive esophagectomy such as video‐assisted surgery can reduce chest wall trauma and has been reported to reduce surgical mortality, preserve pulmonary function and improve patients’ postoperative quality of life.^1^, ^4^, ^5^ However, video‐assisted transthoracic esophagectomy is technically demanding and its perioperative outcome depends on the skill of the surgeon.^6^, ^7^

In an effort to eliminate surgical mortality after esophagectomy, the most essential point is reduction of pulmonary complications. Transthoracic surgery is one of the most important risk factors contributing to the occurrence of pulmonary complications.^8^, ^9^ Even with smaller incisions, transthoracic manipulation mandates one‐lung ventilation or collapsing the right lung with artificial pneumothorax. One‐lung ventilation is reported to result in mechanical damage to both the ventilated and the collapsed lung.^8^, ^10^

To avoid transthoracic manipulation, transhiatal esophagectomy has been a preferred choice. However, the conventional transhiatal approach offers poor surgical view of the mediastinum, especially the upper mediastinum, and mediastinal dissection has been done by blind blunt finger dissections. Therefore, conventional transhiatal esophagectomy is associated with increased recurrent laryngeal nerve injury and inadequate mediastinal lymph dissection.^11^, ^12^ For transhiatal esophagectomy to be a feasible surgery for esophageal malignancies, it should preserve an adequate surgical view of the upper mediastinum.

In such a background, use of the mediastinoscope in the upper retromediastinum has been attempted and mediastinoscopic upper mediastinal dissection has been suggested to be feasible with an excellent surgical view.^13^, ^14^, ^15^, ^16^ Above all, the technique using a single‐incision laparoscopic surgery device described by Fujiwara et al.^15^ was highly reproducible. We have also adopted their technique and developed our understanding of the unfamiliar view of the mediastinum through the mediastinoscope. Hereafter, we describe the details of the video‐assisted transcervical mediastinal dissection (VATCMD) with illustrations and a video. Its perioperative outcomes are also reported.

## 2 MATERIALS AND METHODS

### 2.1 Tasks of VATCMD

Tasks of VATCMD were: (i) en bloc retrieval of lymph nodes in the following stations: upper paraesophageal (#105 nodes), left recurrent laryngeal nerve (#106recL nodes) and left tracheobronchial stations (#106tbL nodes); (ii) complete mobilization of the upper thoracic esophagus; and (iii) division of small vessels at the lateral and dorsal side of the middle esophagus.

### 2.2 Procedures subsequent to VATCMD to complete radical esophagectomy

Cervical incision for VATCMD was extended as a collar incision and the lymph nodes adjacent to the right recurrent nerve (#106recR nodes) and the right side of the esophagus (#101R nodes) were harvested by a right cervical approach under direct vision. Subsequently, conventional laparoscopic followed by transhiatal robotic surgery dissected the abdominal and the middle‐lower mediastinal lymph stations to complete the radical esophagectomy. The specimen was harvested from a 5 cm epigastric incision after transecting the oral margin of the specimen and a cervical anastomosis was carried out for the remnant esophagus and a gastric conduit lifted through the posterior mediastinal route.

### 2.3 Surgical port placement and other preparatory procedures

**Figure 1:**
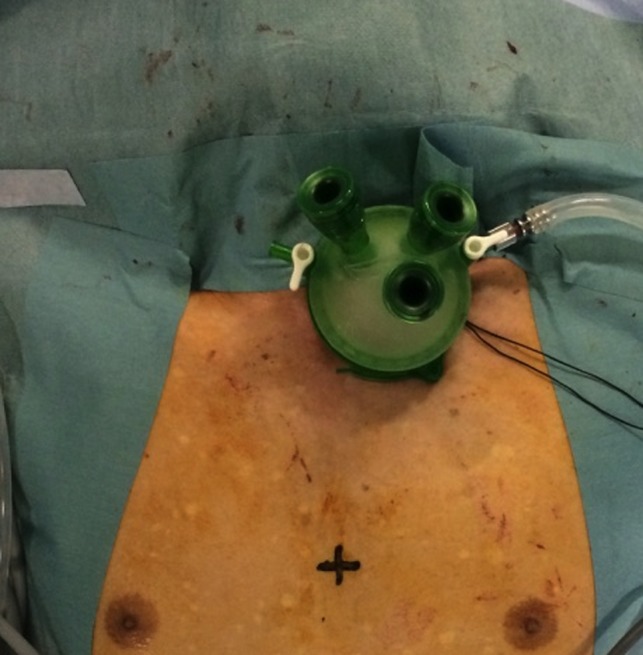
Port placement of video‐assisted transcervical mediastinal dissection (VATCMD). GelPOINT platform (Applied Medical, Rancho Santa Margarita, CA, USA) was placed in a 4 cm left‐sided cervical incision and three ports were placed. The port near the center of the GelPOINT was used for the endoscope and the other two ports were placed for the three ports to form an equilateral triangle.

The patient was placed supine with the head turned to the right. A 4 cm incision was made along the left clavicle. The left half of the strap muscles were incised and the left lateral aspect of the trachea was exposed. By carefully dissecting the tissue located at the left side of the trachea, the left recurrent laryngeal nerve (RLN) was identified and encircled by a small piece of rubber tape. This procedure included retrieval of tissue adherent to the left side of the cervical esophagus (#101L station). Subsequently, the cervical esophagus was dissected from the surrounding tissue and taped by a surgical tape. After these preparations, a device for the single‐port laparoscopic surgery (GelPOINT platform; Applied Medical, Rancho Santa Margarita, CA, USA) was placed to maintain pneumomediastinum by insufflating CO_2_ gas with approximately 8 mmHg pressure (range from 6 to 10 mmHg). Three 5 mm trocars were placed in the single port, one in the center and the other two formed an equilateral triangle. A 5 mm up‐angled 30 degree rigid scope was introduced through the center port (Figure 1) and surgical instruments such as CroceOlmi Grasping Forceps (Olympus, Tokyo, Japan), Fine Maryland Forceps (Olympus), Mini‐Metzenbaum Scissors (Olympus) or LigaSure (Covidien, Norwalk, CT, USA) were inserted through the other two ports placed cranial to the center one. Mainly, the right hand operated the LigaSure, Fine Maryland Forceps and Mini‐Metzenbaum Scissors and the left hand operated the CroceOlmi Grasp Forceps.

### 2.4 Surgical techniques

**Figure 2:**
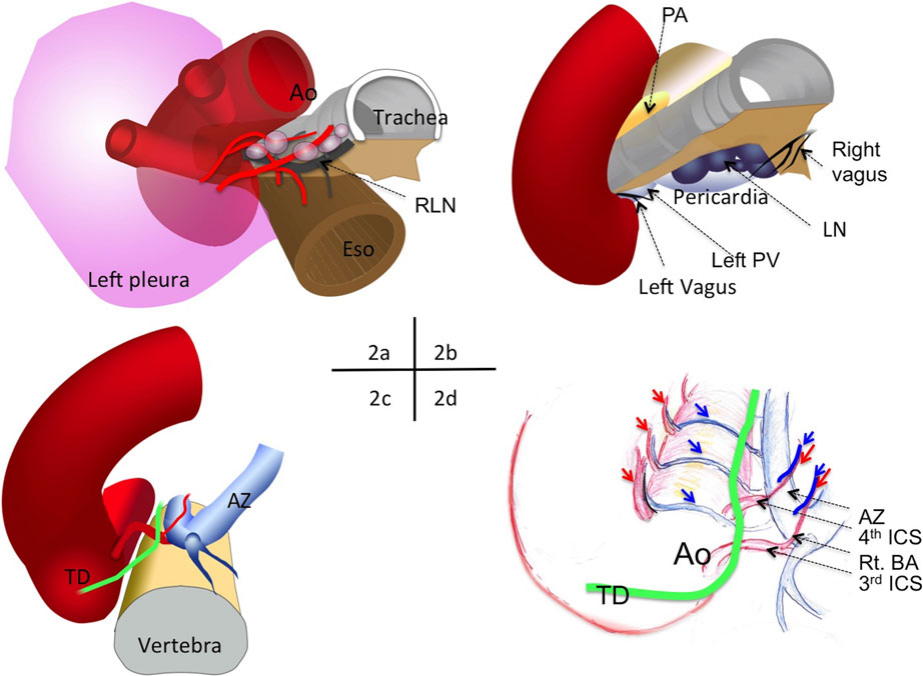
Surgical view of the upper and middle mediastinal anatomies. The esophagus is excluded in these figures except for part (A). (A) Small vessels and nerve fibers distributing to the upper mediastinal structures. Ao, aorta; Eso, esophagus; RLN, recurrent laryngeal nerve. (B) Anatomical structures on the ventral side of the esophagus in the upper‐middle mediastinum. LN, lymph nodes at the subcarinal station; PA, pulmonary artery; PV, pulmonary vein. (C) Middle mediastinal structures dorsal to the esophagus. AZ, azygos vein; TD, thoracic duct. (D) Middle mediastinal structures dorsal to the esophagus after the dissection procedure extended into the more caudal area. The descending aorta, the TD and the AZ are arranged parallel behind the esophagus. ICS, intercostal artery; Rt BA, right bronchial artery. Red and blue arrows, small arteries (red) and veins (blue) distributing to the esophagus. These figures are incorporated into the video to assist comprehension of the video contents.

With a positive insufflation pressure, the connective tissue surrounding the esophagus spontaneously turned into areolar loose tissue (Figure 2; Video S1). First, the left side of the esophagus was dissected from the left pleura and the sheath covering the left carotid artery and the aorta. Dissections along the left‐dorsal side of the esophagus exposed the thoracic duct.

Subsequently, dissections using a bipolar sealer were carried out close to the left side of the trachea dividing small vessel and nerve branches distributing to the trachea. Then, the dissections were carried out for the esophageal attachments to the right pleura and the membrane part of the trachea.

The dissection target was aimed again to the dorsal side of the upper thoracic esophagus where the thoracic duct had been already exposed. Soft and blunt dissection with the tip of a bipolar sealer effectively created a bloodless small operative field behind the esophagus. The space closely in front of the descending aorta usually consisted of loose connective tissue and dissections in this space rarely required bipolar sealing. After these dissections, attachment of the dorsal side of the middle thoracic esophagus was detached with a bipolar sealer and, in nearly all of the cases, this procedure produced a linear sealing trace between the aorta and the azygos vein presumably where the thoracic duct was closely located. These dissections could be easily extended well beyond the fourth right intercostal artery and, in many cases, the proper esophageal arteries arising from the left side of the aorta were observed in a row (Fig. S1). As shown in the video, small retractions by the operator's left hand enabled exposure and dissection of these fine structures without any help from the assistants.

Dividing the esophageal branches of the vagus at the left and right sides exposed such adjacent structures as the peripheral side of the left main bronchus, the left inferior pulmonary vein, the inner aspect of the azygos arch, the right bronchial artery, the dorsal aspect of the tracheal bifurcation and adjacent lymph nodes (#107 nodes).

Finally, dissection along the tracheobronchial angle and the inner aspect of the aortic arch was carried out and the lymph nodes together with the esophagus and the left recurrent laryngeal nerve were isolated from the surrounding mediastinal structures. By isolating the left recurrent laryngeal nerve from the esophagus and the adjacent tissue, the upper mediastinal lymph nodes could be harvested together with the esophagus when the esophageal specimen was retrieved at the latter stage of the surgery. If indicated, the left tracheobronchial nodes (#106tbL nodes) was individually harvested.

### 2.5 Patients

We adopted the devices of the single‐port laparoscopic surgery to VATCMD from August 2015 and, by November 2016, 17 esophageal cancer patients underwent esophagectomy including VATCMD in the format described above. Perioperative outcome of these 17 patients was summarized and reported. All patients gave written informed consent. Indications for non‐transthoracic esophagectomy were: (i) written informed consent to undergo robot‐assisted surgery without receiving financial support from the national health insurance system; (ii) histologically proven esophageal cancer; (iii) sufficiently good general condition to tolerate conventional open esophagectomy; and (iv) a tumor clinically staged as T1‐3 N0‐1 M0 according to the 7th edition of the American Joint Committee on Cancer (AJCC) tumor‐node‐metastasis classification.^17^ Exclusion criteria were: (i) locally advanced cancer with suspicion of invasion to adjacent organs; (ii) prior radiotherapy to the operative field; and (iii) patient aged over 80 years. Clinical characteristics of the patients are summarized in Table 1 and the histological type of all cases was squamous cell carcinoma.

**Table 1:** Characteristics of patients with esophagectomy undergoing VATCMD

Gender (male/female)	16/1
Median age, years (range)	64 (43‐77)
Tumor location (upper/middle/lower/EGJ)	2/11/2/2
Clinical stage	
T factor (1a/1b/2/3)	2/10/5/0
N factor (0/1)	13/4
Prior chemotherapy (yes/no)	0/17

EGJ, esophagogastric junction; VATCMD, video‐assisted transcervical mediastinal dissection.

### 2.6 Perioperative outcomes

Operation time and estimated blood loss, occurrence of procedure‐related intraoperative adverse events, postoperative complications and length of postoperative hospital stay were reported. Operation time for VATCMD phase was measured from the first skin incision to the termination of the transcervical mediastinal insufflation. Blood loss in the VATCMD phase was recorded according to the gauze weight report on completion of the non‐video‐assisted cervical procedure subsequent to VATCMD. Postoperative complications were categorized using a modified Clavien‐Dindo Classification^18^ and complications graded as grade 2 or more were counted in this study. Number of upper mediastinal lymph nodes retrieved by VATCMD and the subsequent non‐video‐assisted cervical procedure was reported.

## 3 RESULTS

In all cases, esophagectomy and mediastinal lymph dissection were completed by VATCMD followed by a right cervical procedure and a laparoscopic‐robotic transhiatal surgery. There was no conversion to the conventional transthoracic esophagectomy and no adverse events closely related to VATCMD were observed. Duration of operation, estimated blood loss of VATCMD and frequencies of postoperative morbidities possibly related to VATCMD are listed in Table 2. Anastomotic leakage was observed in five cases, all of which were cured within 2 weeks by cervical drainage. Median length of postoperative hospital stay was 17 days (range from 11 to 27 days). Median number of lymph nodes retrieved by VATCMD was 10 (range from 2 to 23). All patients underwent curative surgery with pathologically negative circumferential margins.

**Table 2:** Perioperative outcome of patients with esophagectomy who underwent VATCMD

Operative measures	Median (range)
Operative time (min)	
VATCMD	167 (151‐206)
Entire operation	521 (417‐612)
Estimated blood loss (mL)	
VATCMD	20 (10‐30)
Entire operation	215 (20‐690)
Postoperative hospital stay (days)	17 (11‐27)

Postoperative complications	No. cases (%)
RLN palsy	0
Chylothorax	0
Pneumonia	0
Anastomotic leakage	5 (29)

Lymph nodes yield	Median (range)
No. retrieved nodes^a^	10 (2‐23)

RLN, recurrent laryngeal nerve; VATCMD, video‐assisted transcervical mediastinal dissection.

a Total number of upper mediastinal lymph nodes retrieved by both VATCMD and a right cervical procedure.

## 4 DISCUSSION

In VATCMD, the upper mediastinum was close to the cervical skin incision and this proximity reduced the effort required for maintenance of the surgical field. We carried out video‐assisted upper mediastinal dissection using custom‐made retractors in a former series of patients and reported one case (4.5%) of grade 3 RLN palsy in 22 patients undergoing totally non‐transthoracic esophagectomy.^14^ Use of long retractors in a narrow field of the upper mediastinum would not be free from the risk of damaging the left RLN. A device for single‐port laparoscopic surgery could avoid the use of retractors and enabled insufflation of the mediastinum. Pneumomediastinum was very helpful in discovering correct dissection targets considered as loose areolar connective tissue space and bloodless blunt dissections were easily carried out into these loose spaces. These dissections isolated small blood vessels and nerve fibers connecting the esophagus to the adjacent mediastinal structures and they were safely sealed and divided by a bipolar sealer device. In this way, VATCMD was completed without retractors inserted through an additional port for an assistant surgeon. Bloodless surgical field was maintained without difficulty and laborious clean‐up manipulations with gauze or suction were rarely required. Clear visualization of mediastinal structures enabled meticulous dissections close to the left recurrent laryngeal nerve and might contribute to prevention of postoperative recurrent laryngeal nerve palsy.

**Figure 3:**
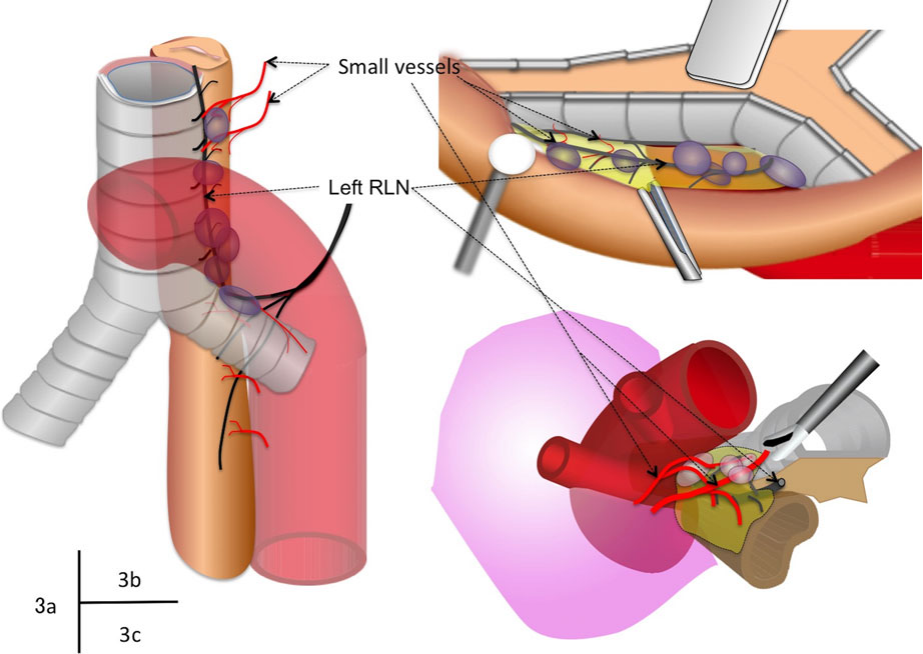
Location of the left paratracheal, left tracheobronchial nodes, adjacent vessels and nerve fibers. (A) Anterio‐left lateral oblique view. Left recurrent laryngeal nerve and lymph nodes are arranged on the opposite side from the right thoracic approach. (B) Surgical view of the right transthoracic approach. Skillful retractions of the trachea and the esophagus are required to expose the lymph nodes and the left recurrent laryngeal nerve. (C) Surgical view of video‐assisted transcervical mediastinal dissection (VATCMD). Lymph nodes were accessible without retractors and a bipolar sealer was placed in a suitable position to dissect small vessels and nerve branches.

Upper mediastinal lymph stations often harbor metastatic disease and the efficacy of the lymph dissections in these stations were reported as high.^19^ However, lymph dissection of the left paratracheal nodes, especially in the higher region, would be the most challenging part of radical esophagectomy through the right transthoracic approach. In this approach, these nodes are located behind the esophagus and the trachea and, therefore, skillful retractions of these bulky anatomies in a narrow surgical field are required to carry out lymph dissection for the paratracheal and tracheobronchial station (Figure 3A,B). In addition, these lymph nodes are closely adjacent to the fine branches of blood vessels and the left recurrent laryngeal nerve. In contrast, VATCMD provided a surgical view in which these branches were viewed without effortful retractions and a sealing device can be easily placed in a suitable position to dissect these branches (Figure 3C).

In summary, VATCMD is suggested as a reasonable and advantageous approach for upper mediastinal dissection in surgery for esophageal malignancies with its accessibility of the left upper mediastinum and its independence of the skill of the assistant. Transthoracic procedures can be shortened or totally omitted with VATCMD incorporated into radical esophagectomy.

## DISCLOSURE

The operative method of VATCMD was part of the surgical procedure of non‐transthoracic robot‐assisted esophagectomy which was approved by a suitably constituted Ethics Committee of the institution (P2011029‐11Y) and it conforms to the provisions of the Declaration of Helsinki. All informed consent was obtained from the subject(s) and/or guardian(s).

Conflict of Interest: Authors declare no conflicts of interest for this article.

## References

[1] Luketich JD , Pennathur A , Awais O , et al. Outcomes after minimally invasive esophagectomy: review of over 1000 patients. Ann Surg. 2012;256:95–103. 22668811 10.1097/SLA.0b013e3182590603 PMC4103614

[2] Osugi H , Takemura M , Higashino M , et al. Video‐assisted thoracoscopic esophagectomy and radical lymph node dissection for esophageal cancer. A series of 75 cases. Surg Endosc. 2002;16:1588–93. 12085146 10.1007/s00464-002-9019-z

[3] Biere SS , Van Berge Henegouwen MI , Maas KW , et al. Minimally invasive versus open esophagectomy for patients with esophageal cancer: a multicentre, open‐label, randomised controlled trial. Lancet. 2012;379:1887–92. 22552194 10.1016/S0140-6736(12)60516-9

[4] Wang H , Feng M , Tan L , Wang Q . Comparison of the short‐term quality of life in patients with esophageal cancer after subtotal esophagectomy via video‐assisted thoracoscopic or open surgery. Dis Esophagus. 2010;23:408–14. 19930404 10.1111/j.1442-2050.2009.01025.x

[5] Taguchi S , Osugi H , Higashino M , et al. Comparison of three‐field esophagectomy for esophageal cancer incorporating open or thoracoscopic thoracotomy. Surg Endosc. 2003;17:1445–50. 12811660 10.1007/s00464-002-9232-9

[6] Takeuchi H , Miyata H , Kitagawa Y , et al. A risk model for esophagectomy using data of 5354 patients included in a Japanese nationwide web‐based database. Ann Surg. 2014;260:259–66. 24743609 10.1097/SLA.0000000000000644

[7] Sudarshan M , Ferri L . A critical review of minimally invasive esophagectomy. Surg Laparosc Endosc Percutan Tech. 2012;22:310–8. 22874679 10.1097/SLE.0b013e3182582d2c

[8] Tandon S , Batchelor A , Bullock R , et al. Peri‐operative risk factors for acute lung injury after elective esophagectomy. Br J Anaesth. 2001;86:633–8. 11575337 10.1093/bja/86.5.633

[9] Canet J , Gallart L , Gomar C , et al. Prediction of postoperative pulmonary complications in a population‐based surgical cohort. Anesthesiology. 2010;113:1338–50. 21045639 10.1097/ALN.0b013e3181fc6e0a

[10] Misthos P , Katsaragakis S , Milingos N , et al. Postresectional pulmonary oxidative stress in lung cancer patients. The role of one‐lung ventilation. Eur J Cardiothorac Surg. 2005;27:379. 15740942 10.1016/j.ejcts.2004.12.023

[11] Boshier PR , Anderson O , Hanna GB . Transthoracic versus transhiatal esophagectomy for the treatment of esophagogastric cancer: a meta‐analysis. Ann Surg. 2011;254:894–906. 21785341 10.1097/SLA.0b013e3182263781

[12] Donohoe CL , O'Farrell NJ , Ravi N , Reynolds JV . Evidence‐based selective application of transhiatal esophagectomy in a high‐volume esophageal center. World J Surg. 2012;36:98–103. 21979584 10.1007/s00268-011-1307-0

[13] Parker M , Bowers SP , Goldberg RF , et al. Transcervical videoscopic esophageal dissection during two‐field minimally invasive esophagectomy: early patient experience. Surg Endosc. 2011;25:3865–9. 21701920 10.1007/s00464-011-1811-1

[14] Mori K , Yamagata Y , Aikou S , et al. Short‐term outcomes of robotic radical esophagectomy for esophageal cancer by a nontransthoracic approach compared with conventional transthoracic surgery. Dis Esophagus. 2016;29:429–34. 25809390 10.1111/dote.12345 PMC5132031

[15] Fujiwara H , Shiozaki A , Konishi H , et al. Single‐port mediastinoscopic lymphadenectomy along the left recurrent laryngeal nerve. Ann Thorac Surg. 2015;100:1115–7. 26354650 10.1016/j.athoracsur.2015.03.122

[16] Mori K , Yoshimura S , Yamagata Y , Aikou S , Seto Y . Preclinical study of transcervical upper mediastinal dissection for esophageal malignancy by robot‐assisted surgery. Int J Med Robot. 2016; https://doi.org/10.1002/rcs.1750. In press. 10.1002/rcs.1750 27273148

[17] Edge SB , editor. AJCC cancer staging manual. 7th ed. New York, NY: Springer; 2010.

[18] Dindo D , Demartines N , Clavien PA . Classification of surgical complications: a new proposal with evaluation in a cohort of 6336 patients and results of a survey. Ann Surg. 2004;240:205–13. 15273542 10.1097/01.sla.0000133083.54934.ae PMC1360123

[19] Tachimori T , Ozawa S , Numasaki H , et al. Efficacy of lymph node dissection by node zones according to tumor location for esophageal squamous cell carcinoma. Esophagus. 2016;13:1–7. 26752982 10.1007/s10388-015-0515-3 PMC4698372

