# Supplementary figures and images for: Technical details of video‐assisted transcervical mediastinal dissection for esophageal cancer and its perioperative outcome
Source: Ann Gastroenterol Surg. 2017 Aug 14;1(3):232–7. doi: 10.1002/ags3.12022 (PMC5881365; doi:10.1002/ags3.12022)

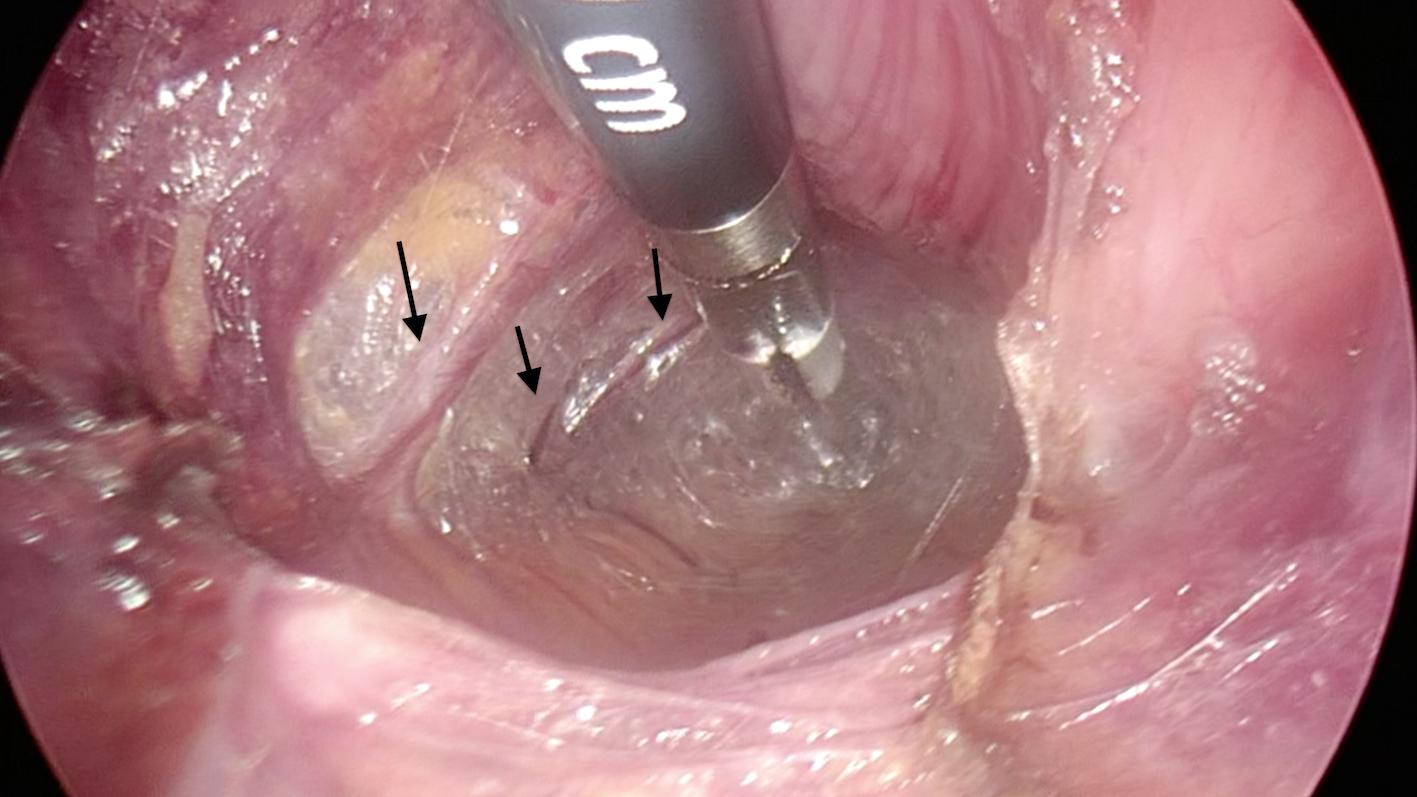

Supplement: Supplementary file 1 [file AGS3-1-232-s001.png]
