# Supplementary material for: Technical details of video‐assisted transcervical mediastinal dissection for esophageal cancer and its perioperative outcome
Source: Ann Gastroenterol Surg. 2017 Aug 14;1(3):232–7. doi: 10.1002/ags3.12022 (PMC5881365; doi:10.1002/ags3.12022)
Supplement: Supplementary file 3 [file AGS3-1-232-s003.docx]

**Video S1**. Surgical technique of VATCMD.

After a Gel-Point platform was placed in the left cervical skin incision and the mediastinum was inflated with CO2 gas, a 5 mm up-angled 30-degree angled rigid scope was introduced through the center port and the provided video was recorded by this scope. The video was explained by the descriptions in “2.4 Surgical techniques.”

**Fig S1**. A operative view in the dorsal side of the middle thoracic esophagus.

Three pairs (arrows) of the proper esophageal arteries and veins are arranged in a low along the frontal side of the aorta. This photo was taken from an operative video of a different surgical case from the one appearing in the Video S1.
